# Association of Visfatin gene polymorphism with obesity related metabolic disorders among Pakistani population: a case control study

**DOI:** 10.1038/s41598-023-48402-z

**Published:** 2023-12-27

**Authors:** Sayyada Humaira Masood, Taseer Ahmed Khan, Akhter Ali Baloch, Syed Muhammad Hasan, Ali Muntazir Naqvi, Mehir un Nisa Iqbal

**Affiliations:** 1 Department of Physiology, University of Karachi, Karachi, Pakistan; 2 Al-Tibri Medical College and Hospital, Isra University Karachi Campus, Karachi, Pakistan; 3 National Institute of Diabetes and Endocrinology (NIDE), Dow University of Health Sciences (DUHS), Ojha Campus, Karachi, Pakistan

**Keywords:** Biotechnology, Bioinformatics, Electrophysiology, Genomic analysis, Sequencing

## Abstract

In recent years, the global prevalence of obesity and its associated metabolic disorders has reached alarming levels, presenting a significant challenge to public health worldwide. Visfatin, also known as pre-B cell colony-enhancing factor (PBEF) or nicotinamide phosphoribosyltransferase (NAMPT), is an adipokine that has been implicated in various physiological processes, including glucose homeostasis, lipid metabolism, and inflammation. The main objective of this proposed study is to find out the association between visfatin genetic variants and metabolic syndrome. The sample size of the study consisted of 300 blood samples (150 control and 150 cases). This study found that the genotypic frequency of visfatin SNPs, including rs2302559 (OD: 18.222; 95% CI 10.228–32.466; *p*-value < 0.001) and rs1215113036 (OD: 129.40; 95% CI 44.576–375.693; *p*-value < 0.001) were significantly associated with metabolic syndrome. Moreover, the frequency of the mutant alleles of both visfatin SNPs was found to be higher in patients with metabolic syndrome as compared to controls. Results of the current study indicate that people with any genetic variation of Visfatin, such as rs2302559 and rs1215113036, are more likely to develop metabolic syndrome. Visfatin genetic variants are linked to an increased risk of metabolic syndrome, implying it’s role in disease pathophysiology.

## Introduction

Metabolic syndrome has been associated with an increased risk of type 2 diabetes, cardiovascular disease, cancer, and all-cause mortality. As plasma-free fatty acid levels increase, they travel to the liver and are potentiated by chronic lipolysis caused by increased adipose tissue bulk^1^. Diabetes, dyslipidemia, high blood pressure, and abdominal obesity are all threats to metabolic syndromes. A high-calorie, high-cholesterol diet paired with a sedentary lifestyle, smoking, drinking alcohol, and gaining weight as an adult are all risk factors for metabolic syndrome, which is linked to obesity. Surprisingly, variations in lifestyle can affect the susceptibility to metabolic syndrome due to genetic variations in insulin resistance and fat distribution^2,3^.

Abdominal obesity and waist circumference are major sponsors of the overall inflammatory state linked with metabolic syndrome, and even people who are minimally overweight but have a high level of abdominal obesity are at risk of unfavorable systemic effects^4^. The adipose tissue's release of different adipokines may be one of the primary mechanisms underlying these lifestyle-related illnesses^5^.

Visfatin is an adipokine released by adipose tissues, and it has been linked to obesity and inflammation in the blood. Visfatin performs various biological functions in the human body, including immunomodulation, catalyzing distinct cellular functions, and anti-apoptosis^6^. Visfatin may be connected to the pathophysiology of diabetes and is essential for the establishment of metabolic syndrome, according to a previous study^5^. The human visfatin gene is located at the 7q22.1 to 7q31.33 chromosome and encodes a protein comprised of 491 amino acids with a molecular weight of 52 kDa. The gene for visfatin has been effectively maintained throughout evolution^7^.

Some single nucleotide polymorphisms (SNPs) for Visfatin that were discovered have been linked to problems associated with obesity and glucose/lipid metabolism. Additionally, improvements in insulin sensitivity and glucose tolerance have both been linked to increases in visfatin concentration in obese patients who have been trained in aerobic exercise. Therefore, Visfatin may be a candidate gene for changes in glucose and obesity-related phenotypes brought on by aerobic exercise training, and its gene polymorphisms may be the reason why different reactions to the same activities are observed in different people^8–10^.

The aim of the current study was to investigate the association of visfatin SNPs, including rs2302559 and rs1215113036, with obesity-related metabolic syndrome.

## Material and methods

Approval of this case–control study was given by IRB of Dow University of Health Sciences (DUHS). (IRB-1969/DUHS/Approval /2021/353). The complete study was carried out in compliance with the relevant guidelines and regulations of the mentioned authority.

This study included 300 participants of of both sexes, with age ranges between 30 to 60 years. All the participants were divided into two groups: the control group (n = 150), comprised of normal healthy individuals with BMI < 23 kg/m^2^^11^, and the case group (n = 150), comprised of overweight/obese individuals (BMI ≥ 23 kg/m^2^)^11^ who are suffering from metabolic syndrome.

Metabolic syndrome was defined following the criteria provided by the modified NCEP ATP III and IDF groups. According to the modified NCEP criteria^12^, the presence of any three of the following five factors is required for a diagnosis of Metabolic Syndrome: abdominal obesity, hypertriglyceridaemia (triglycerides ≥ 150 mg/dL); low HDL cholesterol (HDL cholesterol ≤ 40 mg/dL for men and ≤ 50 mg/dL for women); elevated blood pressure (systolic blood pressure ≥ 130 mmHg and/or diastolic blood pressure ≥ 85 mmHg or current use of antihypertensive drugs); impaired fasting glucose (fasting plasma glucose ≥ 100 mg/dL)^3^.

Individuals suffering from chronic medical conditions like endocrinological problems and cardiovascular and renal diseases were excluded from the study. Written informed consent, along with clinical history, was sought from each participant. All participants of the case group were selected by non-probability purposive sampling technique.

Fasting blood of 6 ml was collected from all participants for biochemical and genetic analysis. Serum Visfatin was evaluated through ELISA techniques while fasting blood glucose analysis and lipid profiling were done by the kit method.

Genomic DNA from whole blood was isolated using a Thersmoscientific DNA isolation kit, which is GeneJET Genomic DNA Purification kit Cat no. K0721. Isolation and purification of DNA was carried out according to the manufacturer’s guidelines.

The genotype distribution of each SNP was checked for the departure from hardy Weinberg equilibrium (HWE) (*p* > 0.05) using the goodness of fit model among controls. HWE > 0.05 indicates that the SNPs donot differ significantly from population.however HWE < 0.05 could show that consanguinity, population structure or problem in genotyping^13^.

Human visfatin SNP rs2302559 anchored position is GRCh38.p14 at chromosome 7. It is a synonymous variant in which the T allele is replaced with A/C/G (NCBI; https://www.ncbi.nlm.nih.gov/snp/rs2302559). In contrast, human visfatin SNP rs1215113036 is a missense variant in which the G allele is replaced with the A allele, and it anchored at GRCh38.p14 at chromosome 7 (NCBI; https://www.ncbi.nlm.nih.gov/snp/rs1215113036). Primers for the Visfatin gene were designed using the online software Primer-1®. The specificity of all the designed primers was confirmed using the online BLAST® program/software. Genotyping of the Visfatin gene’s SNPs (i.e., rs2302559 and rs1215113036) was performed by using tARMs PCR analysis. Conditions for tARMS PCR for both visfatin SNPs are given in Table 1.

**Table 1:** Conditions of tARMS PCR for both SNPs of visfatin gene.

PCR steps		rs 2302559		rs 1215113036	
S. N	Steps	Temperature	Cycles	Temperature	Cycles
1	First hold	94 °C (5 min)	35 Cycles	94 °C (5 min)	35 Cycles
2	Denaturation	94 °C (30 s)		94 °C (30 s)	
3	Annealing	58.25 °C (30 s)		62 °C (35 s)	
4	Elongation	72 °C (30 s)		72 °C (30 s)	
5	Second hold	72 °C (7 min)		72 °C (7 min)	
6	Storage	4 °C (∞)		4 °C (∞)	

Fragments obtained after amplification were analyzed using agarose gel electrophoresis, and band sizes of selected SNPs were compared with a 100 bp DNA ladder. The confirmation of DNA sequences of both Visfatin SNPs was done using direct DNA Sequencing, and the sequencing file was analyzed with Mega11 software.

All statistical analyses were performed using SPSS software (SPSS Inc. version 20, Chicago, IL, USA). The distribution of demographic variables between the case group and controls was done by the Kolmogorov–Smirnov (KS) test. At the same time, mean differences of continuous variables between case and control groups were done by independent sample t-test. The Pearson's correlation were used for anthropometric, fasting blood glucose and lipid profile parameters with serum visfatin. Chi-square was used to determine the genotypic frequency between both groups. Relative associations between genotypes of selected visfatin SNPs and both groups (i.e., cases and controls) were assessed by calculating the odds ratios and 95% confidence intervals. *P*-value < 0.05 was considered statistically significant.

## Results

### Demographic characteristics of MetS patients and controls

Demographic information was gathered and collected from patients with metabolic syndrome caused by obesity. MetS was significantly associated with age groups in the context of gender and age groups. In contrast, in individuals with HTN, obesity, and diabetes, MetS was found to be significantly (*p*-value < 0.001) linked with medical history. Positive family history were found significant in individuals with metabolic syndrome, as shown in Tables 2 and 3.

**Table 2:** Demographic characteristics of MetS patients and controls.

Groups	Controls		Cases		t statistics	
	Mean	± SD	Mean	± SD	Value	*P*-value
Age Years	37.90	8.946	47.53	9.183	− 9.196	< 0.001*

SD Standard Deviation, *Statistically significant.

**Table 3:** Demographic Characteristics of MetS Patients and Controls.

Group		KS statistics	
		Value	*P*-value
HTN			
Yes	150	0.461	< 0.001*
No	150		
Diabetes			
Yes	150	0.341	< 0.001*
No	150		
Obesity			
Yes	150	0.476	< 0.001*
No	150		
Family history			
Yes	156	19.74	< 0.001*
No	144		

KS Kolmogorov–Smirnov Test, HTN hypertension; *statistically significant.

### MetS association with anthropometric measurements and general physical parameters

In Table 4, it is shown that individuals with metabolic syndrome had significantly (*p*-value  <0.001) higher levels of weight, body mass index, systolic and diastolic blood pressure, and pulse rate when anthropometric and general physical parameters were compared between the two groups.

**Table 4:** MetS association with anthropometric measurements and general physical parameters.

Groups	Controls		Cases		t-test	
Statistics	Mean	SD	Mean	SD	T	*P*-value
Weight (kg)	63.73	6.879	74.66	16.172	− 7.615	< 0.001*
Height (m)	1.6702	0.08577	1.6631	0.07904	0.745	0.457
BMI (Kg/m^2^)	22.8517	1.89748	27.0099	5.62306	− 8.582	< 0.001*
Mid arm circumferences (in)	12.2807	1.12731	12.1127	0.83145	1.469	0.143
Systolic blood pressure	119.98	8.180	132.91	17.188	− 8.317	< 0.001*
Diastolic blood pressure	76.40	6.801	79.99	10.305	− 3.558	< 0.001*
pulse	78.93	7.202	81.80	5.531	− 3.866	< 0.001*

SD, standard deviation; BMI, body mass index; *statistically significant.

### MetS patient’s biochemical analysis

Biochemicals, including serum visfatin, serum cholesterol, triglycerides, LDL and VLDL cholesterol, and fasting blood sugar, were found to be significantly (*p*-value < 0.01) higher in patients with MetS as compared to controls, as shown in Table 5.

**Table 5:** Biochemicals Analysis of Patients with MetS.

Groups	Controls		Cases		t-test	
Statistics	Mean	SD	Mean	SD	T	*P*-value
Serum visfatin	2.8489	1.23514	10.0777	5.13731	− 16.756	< 0.001*
Fasting blood sugar	92.9667	15.59617	231.1733	83.91437	− 19.832	< 0.001*
Serum cholesterol	174.27	16.281	184.97	28.539	− 3.989	< 0.001*
Triglycerides	119.35	16.335	126.33	23.967	− 2.950	< 0.001*
LDL C	92.25	12.487	99.03	19.613	− 3.568	< 0.001*
VLDL C	23.98	4.281	25.49	4.128	− 3.103	0.002*
HDL C	48.03	6.110	47.79	6.892	0.328	0.743

SD, standard deviation; *statistically significant; LDL C, low-density lipoprotein cholesterol; VLDL C, very low-density lipoprotein cholesterol; HDL C, high-density lipoprotein cholesterol.

### Serum Visfatin correlation with anthropometric,general physical and biochemical factors

In contrast to patients, results from Pearson correlation analysis indicated a significant (*p*-value < 0.001) negative correlation between MetS and diastolic blood pressure in healthy individuals. Overall findings revealed a significant (*p*-value < 0.001) positive correlation between visfatin and several variables, including age, weight, BMI, systolic blood pressure, pulse rate, fasting blood sugar, serum cholesterol, triglycerides, and LDL cholesterol, as shown in Table 6.

**Table 6:** Serum Visfatin correlation with anthropometric, general physical and biochemical factors.

Correlation	Overall group	
	correlation coefficient	*P*-value
Serum visfatin (ng/mL)	1	
Age	0.275**	< 0.001*
Weight	0.294**	< 0.001*
Height	− 0.007	0.900
BMI (Kg/m^2^)	0.306**	< 0.001*
Mid-arm circumferences	− 0.037	0.524
SBP	0.253**	< 0.001*
DBP	0.109	0.058
Pulse	0.145*	0.012*
FBS	0.555**	< 0.001*
Serum cholesterol	0.205**	< 0.001*
Triglycerides	0.126*	0.029*
LDL C	0.180**	0.002*
VLDL C	0.098	0.089
HDL C	0.036	0.538

*Statistically significant (< 0.05).

### Allele and genotype frequency of visfatin SNPs (rs2302559, rs1215113036)

In patients with MetS and healthy persons, the allele and genotype frequencies of the visfatin gene and its SNPs were also determined. In patients with MetS compared to controls, the homozygous mutant genotype frequency, or CC, for the visfatin SNP rs2302559 was found to be 18-fold higher (OR = 18.222; 95%CI = 10.228–32.466). However, compared to a normal person, MetS patients had a significantly (*p*-value < 0.001) higher frequency of the mutant allele, C, as mentioned in Table 7. Hardy Wingeberg Equation demonstrate the consistency in the gene pool in Tables 7 and 8.

**Table 7:** Hardy Weiberg equilibrium for normally distributed data.

SNP	Genotype	Observed (n)	Expected (n)	Chi square = x^2^	*p*-value
rs2302559	TT	00	25.2	72.43	0.0000
	TC	123	72.6		
	CC	27	52.2		
Rs 1215113036	GG	117	118.2	2.29	0.1300
	GA	33	29.4		
	AA	00	1.8		

*P value > 0.05 indicates non significant results which showed consistency with HWE.

**P value < 0.05 indicates significant results and deviation of genotype distribution from HWE.

**Table 8:** Allele and Genotype Frequency of Visfatin SNPs (rs2302559 and rs1215113036).

Polymorphism	Control	Cases	Chi square	*P* value	OR	(95% CI)		*p*-value	Allele Frequencies	
						Lower	Upper			
rs2302559	n (%)	n (%)							n	%
Allelic frequency										
T	123 (41.0%)	30 (10.0%)			1				153	25.5
C	177 (59.0%)	270 (90.0%)			6.45	4.02	9.72	< 0.001*	447	74.5
Genotypic frequency										
TC	123(82.0%)	30(20.0%)	115.36	0.000	1					
CC	27(18.0%)	120(80.0%)			18.222	10.228	32.466	< 0.001*		
rs1215113036										
Allelic frequency										
G	267 (89.0%)	150(50.0%)			1				417	69.5
A	33 (11.0%)	150(50.0%)			8.09	5.28	12.39	< 0.001*	183	30.5
Genotypic frequency										
GG	117 (78.0%)	4(2.7%)	176.86	0.000	1					
GA	33 (22.0%)	146(97.3%)			129.40	44.576	375.693	< 0.001*		

n—number of samples; %—percentage of samples; OR—ODD ratio; 95%CI—confidence interval; *—statistically significant.

**Figure 1:**
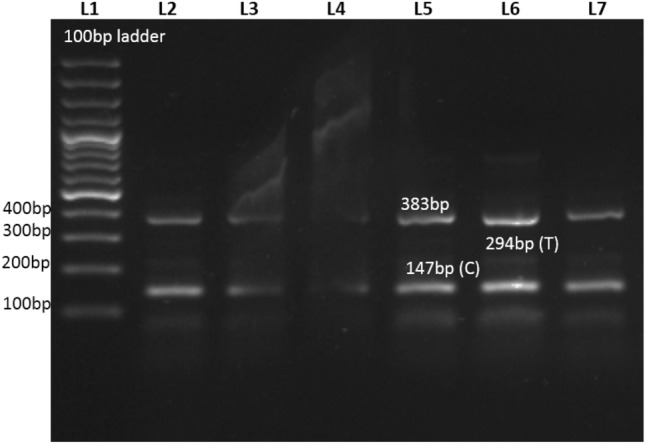
Representative Gel Image of tARMS PCR of rs2302559. L1 = ladder, L2,L3,L4 = controls , L5, L6 and L7 = patients.

Amplification results of visfatin rs2302559 SNP showed the homozygous mutant (CC) with 383 bp and 147 bp bands and heterozygous (CT) genotypes with 383 bp, 294 bp, and 147 bp bands, as demonstrated in Fig. 1.

Additionally, it was discovered that individuals with MetS had a 129-fold higher frequency (OR = 129.40; 95% CI = 44.576–375.693) of the heterozygous mutant genotype, or GA, for the visfatin SNP rs1215113036 than did the controls. The frequency of the mutant allele, A, was significantly (*p*-value < 0.01) higher in MetS patients when compared to controls, as mentioned in Table 7, rs rs2302559 showed concsistency with Hardy Weinberg equation (HWE) while rs1215113036 deviated from HWE as showed in Table 8.

**Figure 2:**
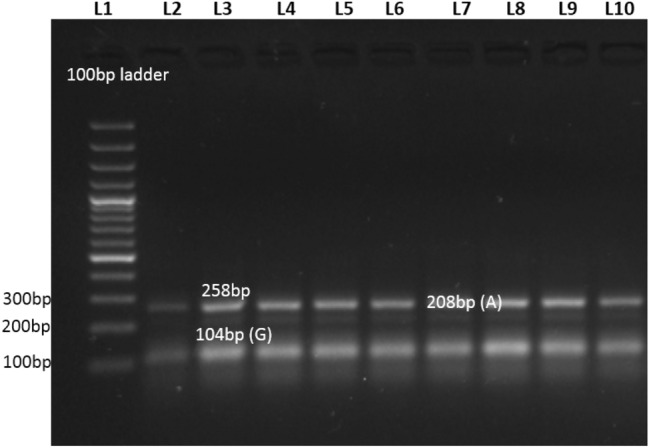
Representative Gel Image of tARMS PCR of rs1215113036.

However, amplification results of visfatin rs1215113036 SNP showed the wild type (GG) genotypes with 258 bp and 104 bp bands and heterozygous (GA) genotypes with 258 bp, 208 bp, and 104 bp bands, as demonstrated in Fig. 2.

**Figure 3:**
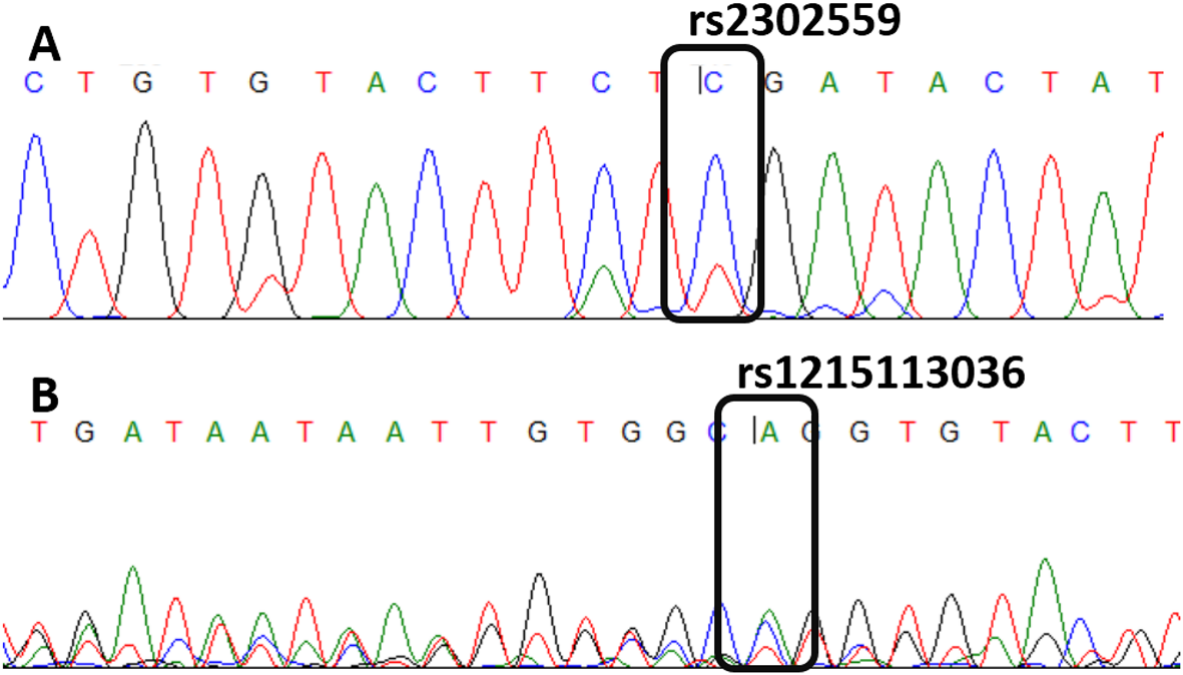
Sequence alignment with wild-type sequence.

Sequencing results showed the complete alignment of visfatin gene sequence of selected samples with the wild-type sequence of visfatin gene. Targeted sequences of visfatin SNPs including rs2302559 and rs1215113036 along with some other variations were also confirmed through sequencing analysis as demonstrated in Fig. 3A and B respectively.

## Discussion

The World Health Organization defines human obesity as an excessive buildup of adipose tissue (adiposity), which constitutes a body mass index (BMI) > 30 kg/m^2^. The metabolic syndrome, which includes hyperglycemia, insulin resistance, and dyslipidemia, can be thought of as a group of issues brought on by obesity^1–3^. The development of chronic diseases like type 2 diabetes (T2D) mellitus, non-alcoholic fatty liver disease (NAFLD), and cardiovascular disease (CVD) is supported by the low-grade inflammation known as metabolic syndrome. A high-fat diet is used to imitate parts of metabolic syndrome in mice, where 40–60% of caloric intake comes from dietary fat. This results in diet-induced obesity and related metabolic abnormalities, such as insulin resistance, as well as concomitant low-grade inflammation^14–16^.

The main emphasis of the present study was to investigate the association of visfatin SNPs, including rs2302559 and rs1215113036, with obesity-related metabolic syndrome in local population of Pakistan.

MetS prevalence in morbid obesity increased in both sexes after the age of 54. However, the percentage of women increased dramatically after this age, compared to a more modest increase in the same percentage of men^17–21^. Earlier studies indicated that those with a positive family history of hypertension had a considerably greater prevalence of hypertension. Additionally, the prevalence of obesity, central obesity and metabolic syndrome is linked to family history of hypertension. These investigations confirmed our findings, which point to a strong correlation between age and a positive family history in patients with metabolic syndrome^22^.

Insulin resistance is a significant factor in the pathophysiology of the metabolic issues associated with visceral obesity. Insulin activity is thought to be influenced by adipokines; chemical messengers generated by adipocytes. In metabolic disorders, the complex chemical visfatin circulates at higher quantities. In a prior study, it was discovered that obese MetS cases has visfatin levels that are significantly higher than those of controls and MetS people who are not obese. Additionally, visfatin serum concentration increases with increasing BMI and has a favorable correlation with lipid parameters, waist circumference, and BMI. Previous studies have also shown that MetS sufferers had higher amounts of visfatin than controls similarly in our study elevated visfatin level in MetS patients were observed^23–25^.

Several physical factors have been found to be strongly linked to metabolic syndrome, according to research. Weight and BMI (Body Mass Index) are important indicators of metabolic syndrome as excess weight is a significant risk factor for developing this condition. Biochemicals such as serum visfatin, triglycerides, fasting blood sugar, serum cholesterol, LDL (low-density lipoprotein) and VLDL (very low-density lipoprotein) cholesterol have also been found to be significant in metabolic syndrome^10^. Age, BMI, blood pressure, fasting blood sugar, triglycerides, serum LDL cholesterol have all been found to positively correlate with metabolic syndrome^26^. Another study reported Metabolic syndrome has been found to have a positive correlation with age, BMI, blood pressure, fasting blood sugar, triglycerides, serum cholesterol, and LDL cholesterol^27^. In some studies, visfatin found to be associated with cardiovascular metabolic disorders (31). These findings are consistent with our study which revealed a significant (*p*-value < 0.001) positive correlation between visfatin and several variables, including age, weight, BMI, systolic blood pressure, pulse rate, fasting blood sugar, serum cholesterol, triglycerides, and LDL cholesterol. This means that as these factors increase the likelihood of developing metabolic syndrome. Therefore, it is essential to maintain a healthy weight, manage blood pressure, and control blood sugar and lipid levels to prevent the development of metabolic syndrome and reduce the risk of developing cardiovascular disease and type 2 diabetes.

The analysis of these two SNPs, rs2302559 and rs1215113036, demonstrates different patterns of deviation from Hardy–Weinberg equilibrium. For rs2302559, there is a significant departure from HWE, indicating that some factors, such as selection or genetic drift, are likely influencing the distribution of genotypes. Further research and investigation are necessary to determine the underlying causes. On the other hand, rs1215113036 appears to be in HWE, as there is no strong evidence to suggest a departure from equilibrium. This suggests that the allele frequencies for this SNP are stable and not subject to significant evolutionary pressures in the studied population. HWE analysis is a valuable tool in population genetics to detect departures from the expected genotype frequencies^28^. The observed deviations from HWE for rs2302559 highlight the need for further investigation into the factors driving this departure, while the adherence to HWE for rs1215113036 suggests genetic stability for this SNP within the population under study. Understanding these genetic patterns can provide insights into evolutionary forces at play in populations and their potential health implications.

Previous investigations have proposed that visfatin may be regulated by insulin, and that visfatin and insulin are more strongly linked than visfatin and glucose. This suggests that visfatin may play a role in glucose metabolism and insulin sensitivity. It is also noted that there have been conflicting findings regarding the relationship between visfatin expression and insulin resistance. This suggests that some studies have found a strong association between visfatin and insulin resistance, while others have not found any significant association^7,29,30^. A potential link between serum visfatin levels and obesity was found. This implies that obesity could also impact the expression and activity of visfatin in the body, which could potentially affect insulin sensitivity and glucose metabolism^10,17,31,32^. One possible explanation for these inconsistent findings is the influence of gene variations involved in the production or function of visfatin and insulin. Genetic variations can impact the expression and activity of hormones in the body, and this may explain why some studies have found a strong association between visfatin and insulin resistance, while others have not.

The statement refers to several studies that have investigated the relationship between different genotypes of Visfatin and various anthropometric measurements, such as BMI, waist circumference, waist-to-height ratio, and fat mass. One study found no significant differences between the genotypes of Visfatin rs2302559 and these anthropometric measurements^31^. In another study, Visfatin levels were not significantly different between SNP rs2302559 genotypes. However, a different study found that carriers of the rs2302559 variant allele had lower fasting serum visfatin and fasting blood glucose levels than wild-type allele carriers in obese children^33^. Notwithstanding the results of the current study differ from these previous findings. Specifically, the results of the current study indicate that people with any genetic variation of Visfatin, such as rs2302559 and rs1215113036, are more likely to develop metabolic syndrome.

The study done by Javanmard et al., (2016) had investigated whether G-948T(rs2302559) gene polymorphism has an association with obesity and comorbidities such as dyslipoproteinemia in Iranian population. Their findings suggested that variations in this polymorphism were correlated with obesity, total cholesterol, and LDL-C levels in carriers of T allele. This study results were similar to our study^34^. Increased BMI is associated with an increased risk of developing high blood pressure, high blood sugar levels, and abnormal lipid levels, which are all components of metabolic syndrome. Blood pressure is another important physical factor that is strongly linked to metabolic syndrome^34^. High blood pressure can damage blood vessels and increase the risk of developing cardiovascular disease. Elevated pulse rate is also an important factor that is often associated with high blood pressure and metabolic syndrome.

The fact that genetic variations in Visfatin are associated with an increased risk of metabolic syndrome suggests that Visfatin may play a role in the development of these conditions. There have been conflicting findings in previous studies regarding the relationship between different Visfatin genotypes and various anthropometric measurements and metabolic parameters. However, the results of the current study suggest that genetic variations in Visfatin may increase the risk of developing metabolic syndrome.

## Conclusion

Metabolic syndrome is characterized by multiple factors, including central obesity, dyslipidaemia, hypertension, and impaired glucose tolerance. Most significant aspect of metabolic syndrome is thought to be obesity. Influence of gene variants involved in the synthesis or action of these hormones could also explain the conflicting results when considering the relationship between serum visfatin levels and obesity. However, there is a lot of debate and very little research on these polymorphisms. Even though the SNPs for visfatin rs2302559 and rs1215113036 were found to be linked to metabolic factors in this study, further research is needed to figure out how exactly the studied SNPs affect metabolic mechanism. In order to complete the profile of these polymorphisms and confirm the association at the populational level, additional genetic studies in larger study groups are required.

## Data Availability

The datasets used and analysed during the current study available from the corresponding authors on reasonable request.
